# Effect of a stranded hole type on the performance of corn stover composite pipe

**DOI:** 10.1371/journal.pone.0301590

**Published:** 2024-04-10

**Authors:** Jie Yang, Yang Guan, Dongdong Gu, Yuzhong Zhang, Zheng Zhang, Jinfa Shi

**Affiliations:** North China University of Water Resources and Electric Power, Zhengzhou, 450046, China; The Energy Resources Institute (TERI), INDIA

## Abstract

To promote the comprehensive utilization of corn stover and the development of field water-saving irrigation technology, a method of returning corn stover to the field was prosed; in this method, the crop stalks were crushed, mixed with soil in different proportions of adulteration, and then extruded to form hollow round tubes. To compare the influence of the winch blade with or without a diameter change on the composite pipe molding performance, two composite pipe molding devices were theoretically designed, simulated, and analyzed using discrete element simulation software, and a composite pipe molding bench test was performed. The simulation test revealed that the composite pipe molding rate of the winch blade without the reducer molding device was 3.45 kg/s, the output power of the winch shaft was 20.7 kW, the composite pipe molding rate of the winch blade with the reducer molding device was 1.20 kg/s, and the output power of the winch shaft was 18.75 kW. By calculating the weighted average of two indices, the composite pipe forming rate and the winch shaft output power, the comprehensive performance index of the composite pipe forming device without a reducer was greater than that of the device with a reducer. The composite pipe forming bench test revealed two kinds of molding devices with an extrusion molding with an outer diameter of 100 mm and an inner diameter of 30 mm. The composite pipe density test average was greater than 1.30 g/cm^3^ and met the requirements of composite pipe molding; the winch blade without a reducer molding device had an average composite pipe molding rate of 3.23 kg/s, and the winch blade with an average reducer molding rate of 2.07 kg/s. The forming rate of the composite pipe without a reducer was faster. Therefore, a winch blade without a reducer composite pipe molding device is more conducive to improving the composite pipe molding performance.

## Introduction

At present, China’s corn stover is mainly concentrated in the Huanghuaihai region. To effectively solve the problem of stover burning, China has vigorously promoted the mechanization of corn stover return technology. Straw farming improves soil fertility, increases the content of soil organic matter, and makes the land more fertile [1–3]. Returning straw to the field is conducive to enhancing soil health, promoting the growth of beneficial flora by increasing soil organic matter content, improving soil structure and function, etc. The relevant flora can participate in the decomposition and maturation of straw to improve soil fertility, reduce the amount of chemical fertilizers, enhance the capacity for carbon sequestration and emission reduction in the farming system, and effectively avoid the problems of soil nodulation as well as serious soil pollution and degradation [4–8].

Mechanized field equipment for corn stover mainly includes whole straw and straw crushed surface covering field equipment, mixed buried field equipment, turning buried field equipment, crushed ditch buried field equipment, and crushed ditch buried field equipment. The corn stover crushing furrow buried field equipment uses the straw and soil bonding effect to maintain a stable granular structure. By covering the straw for soil moisture supplementation and, preventing the soil moisture from evaporating too quickly, the organic matter in corn stover and the soil nutrients such as nitrogen, phosphorus, potassium, and other nutrients can carry out a series of complex biochemical reactions, generating organic nitrogen salts and organic phosphates, and promoting the absorption of nitrogen, potassium, and other nutrients by the root system of the crop and improving the yield and quality of the crop [9,10].

However, corn straw furrow burying technology is still in the research and development stage. Yulun Chen [11] designed a rice and wheat combine harvester and furrowing machine as the object of research and then designed a rice and wheat combine harvesting furrow burying multifunctional integrated machine. The operation could be completed in the rice and wheat crop by combining harvesting, furrowing, and putting straw into the furrow to return to the field and other processes. Degang Kong et al. [12] designed a screw-type straw deep applicator monoblock device based on the requirements of straw deep application to return to the field by drawing on the structure of a deep pine shovel. At present, in terms of single operations, the basic mechanization requirements of furrowing, mulching, and suppression operations can be reached, but the straw burying operation needs to be manually carried out. In terms of joint operation, a more mature integral machine overall operation system has not yet been formed, and the work of the whole machine system needs to be optimized to improve work performance and efficiency. In addition, straw furrow burying and field return joint operation machines can also be considered at the same time to increase the application of nitrogen fertilizer, preservatives, and other functions.

To promote the development of corn stover furrow burying and field return technology and field water-saving irrigation technology and based on the theory of circular bioeconomy, the biocycling economy refers to an economic model that utilizes biomass resources, converts these resources into energy, chemicals, and materials through biochemical and bioprocessing technologies, and recycles them in the industrial chain. Different from the linear economic model in the traditional sense, the biocycling economy focuses on resource saving and recycling to maximize the use of resources. In the biocyclic economy, biomass can be converted in different ways to obtain different products, such as energy, chemicals, and materials, and can play a role in different application fields [13,14].

The method of returning corn stover to the field using extruded corn stover to form hollow round tubes is a sustainable solution in which the crop stover is crushed and mixed with the soil in varying proportions and extruded to form hollow round tubes. The computations were designed for two kinds of composite pipe forming devices: (1) a composite pipe forming device with a stringer blade diameter and (2) a molding device that does not change the diameter of the composite pipe molding density and molding rate. The test results were analyzed using discrete element simulation software, and a bench test was used to compare and study the effect of a stringer blade with or without a diameter change on the composite pipe molding performance, to determine the optimal corn stover molding device.

## Structural design and principle

### Forming device with no reducer for winch blades

**Fig 1:**
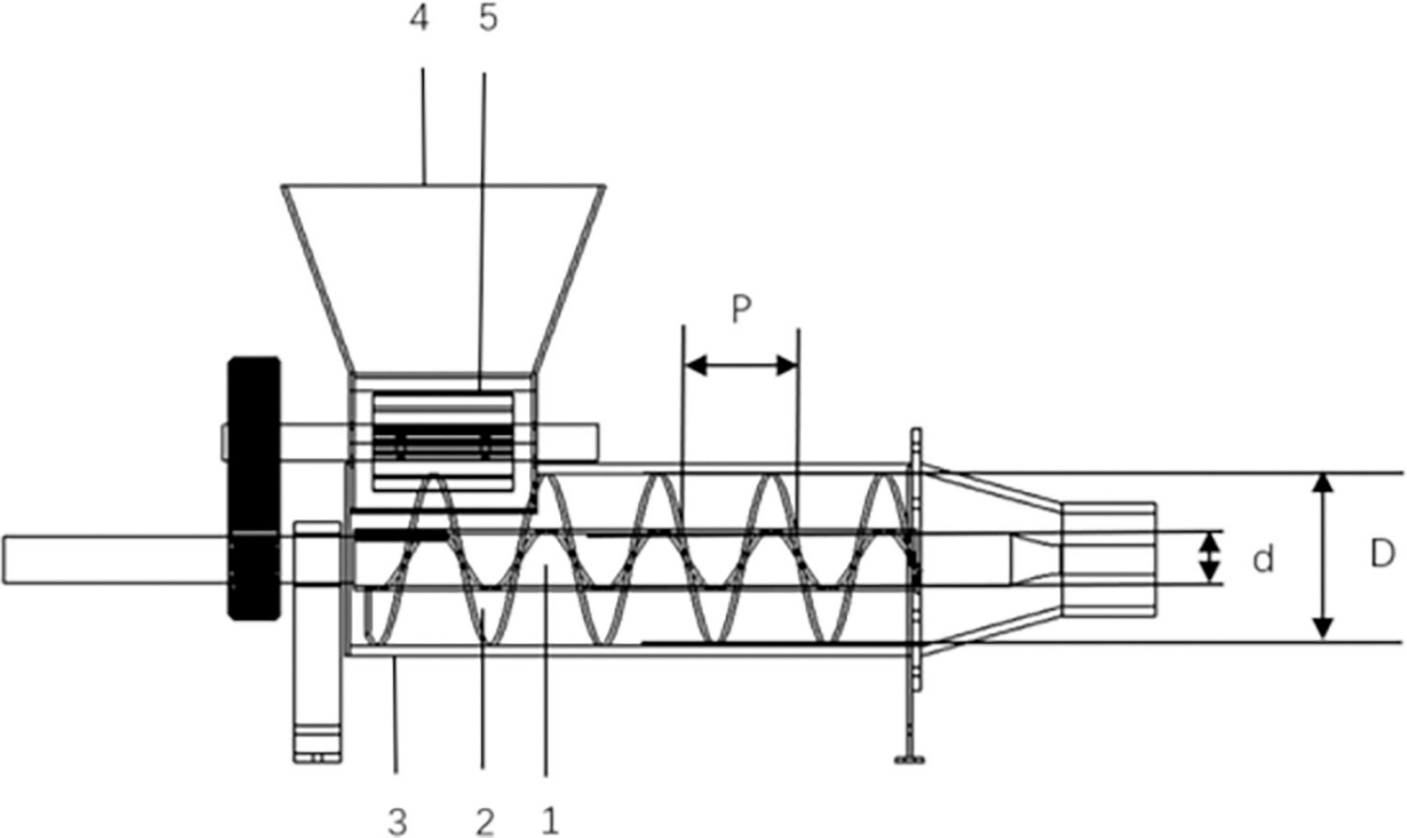
Forming device with no change in the diameter of the winch blades. 1. winch shaft, 2. winch blades, 3. shell, 4. feed cylinder, and 5. mixing plate.

The forming device mainly consists of a winch shaft and winch blades. The shell is both a protective structure and a major component of the straw composite tube shaping. When the composite pipe-forming device began normal operation, a certain proportion of straw and soil was mixed, and water was added to ensure that the moisture content of the mixture of the material reached a certain value. The viscosity of the mixture increased to facilitate compression into a pipe. The mixed straw and soil entered the feed cylinder, and the mixing plate was mixed twice. Under the drive and extrusion of the winch shaft and winch blades, as well as the joint action of the shell, a composite pipe was formed. The structure of the molding device with no change in the diameter of the winch blades is shown in Fig 1. The front end of the winch shaft has no winch blade for the purpose of forming a hollow round tube, which can be filled with water to facilitate drip irrigation of the next crop. After compression molding, the mixture was further compressed in the discharge cylinder; the discharge cylinder was connected to the shell by a flange for easy disassembly and could be replaced with other discharge cylinders having different sized inner diameters to extrude and mold the corn stover composite tubes with different sized outer diameters.

The screw diameter affects the size of the winch shaft diameter; the winch shaft diameter and the screw diameter together affect the angle of rise of the winch blades, which also determine the speed and direction of movement of the mixture in the winch conveyor. The forming speed of the composite pipe can be improved by determining a suitable combination of parameters for the diameter of the winch blade (D), the diameter of the winch shaft (d), and the pitch of the winch (P).

As an important parameter of extrusion molding components, the diameter of the winch blades directly affects the size and output of the device. The determination of the diameter of the winch blades needs to consider factors such as the type of material conveyance, production capacity, layout, and structure of the extrusion molding unit components.

The formula for the diameter of the winch blade as follows [15]:

$$D=K{\left(\frac{Q}{\varphi \lambda \varepsilon }\right)}^{\frac{2}{5}}$$ (1)

Eq: *K‒*Coefficient of the integrated material properties, with a value of 0.05;

Q‒Displacement per unit time of the winch conveyor;

φ‒Filling factor;

λ‒Material density; and

ε‒Inclined transport coefficient.

To ensure that the feed rate of the mixture from the extrusion molding unit is consistent with the discharge rate of the straw composite tube, the discharge per unit of time of the strand conveyor should be equal to the feed rate. The field test was conducted according to GB/T24675.6–2009 "Protected Tillage Machinery Straw Crushing and Returning Machine". Before calibration, a distance of 10 meters was measured with a measuring tape, and a driving distance of 15 meters was measured. The driving speed needed to be a fixed value before entering the test site; after entering the 10-meter measurement zone, the running time of the unit was determined by using a stopwatch, and the tractor’s traveling speed was obtained in the end. The test showed that the amount of straw picked up by the straw returner with a width of 100 cm for every 100 cm forward was 1394.72 g, and since the amount of straw picked up accounted for 5% of the total amount of material, the total mass of the mixture could be calculated to be 27894.40 g. According to the average traveling speed of the tractor, the amount of material fed was determined to be Q = 4.5 t/h. Substituting D into Formula (1): yields *$D=0.05\times {\left(\frac{Q}{0.3\times 0.15\times 0.46}\right)}^{\frac{2}{5}}$*, specifically, D = 0.430 m = 430 mm.

The screw diameter affects the size of the winch shaft diameter; the winch shaft diameter and screw diameter together affect the angle of rise of the winch blade, which also determine the speed and direction of movement of the mixed material in the screw winch conveyor. Therefore, a reasonable relationship between the value of the winch shaft diameter and screw diameter needs to consider the friction between the spiral surface of the winch blade and the extruded material conveyed and the velocity component of the mixed material.

In general, the formula for calculating the shaft diameter of the stringer is *d* = (0.2~0.35)*D*. The value is 0.2, hence, d = 86 mm. The screw gibbet pitch not only affects the spiral lift angle but also affects the slip surface of the material transported under a certain filling coefficient; thus, the size of the pitch directly affects the state of mixed material transport [16,17]. When the displacement per unit time of the strand conveyor Q and the diameter of the winch blade D have a determined value, the strand pitch changes, the mixture of material transportation slip surfaces will also change, and the corresponding mixture of the material transportation speed distribution changes. Therefore, the determination of the gibbet pitch needs to meet the following two conditions: (1) the distribution relationship between the components of the material transportation speed and (2) the friction relationship between the winch blade surface and the mixture of materials.

The formula for calculating the winch pitch is S = K_1_ D. Usually, the value of K_1_ ranges from 0.8 to 1.0 [18,19]. In this study, K_1_ is 0.9, and substituting this value into the formula yields S = 387 mm.

### Forming device for reducing the diameter of the winch blades

**Fig 2:**
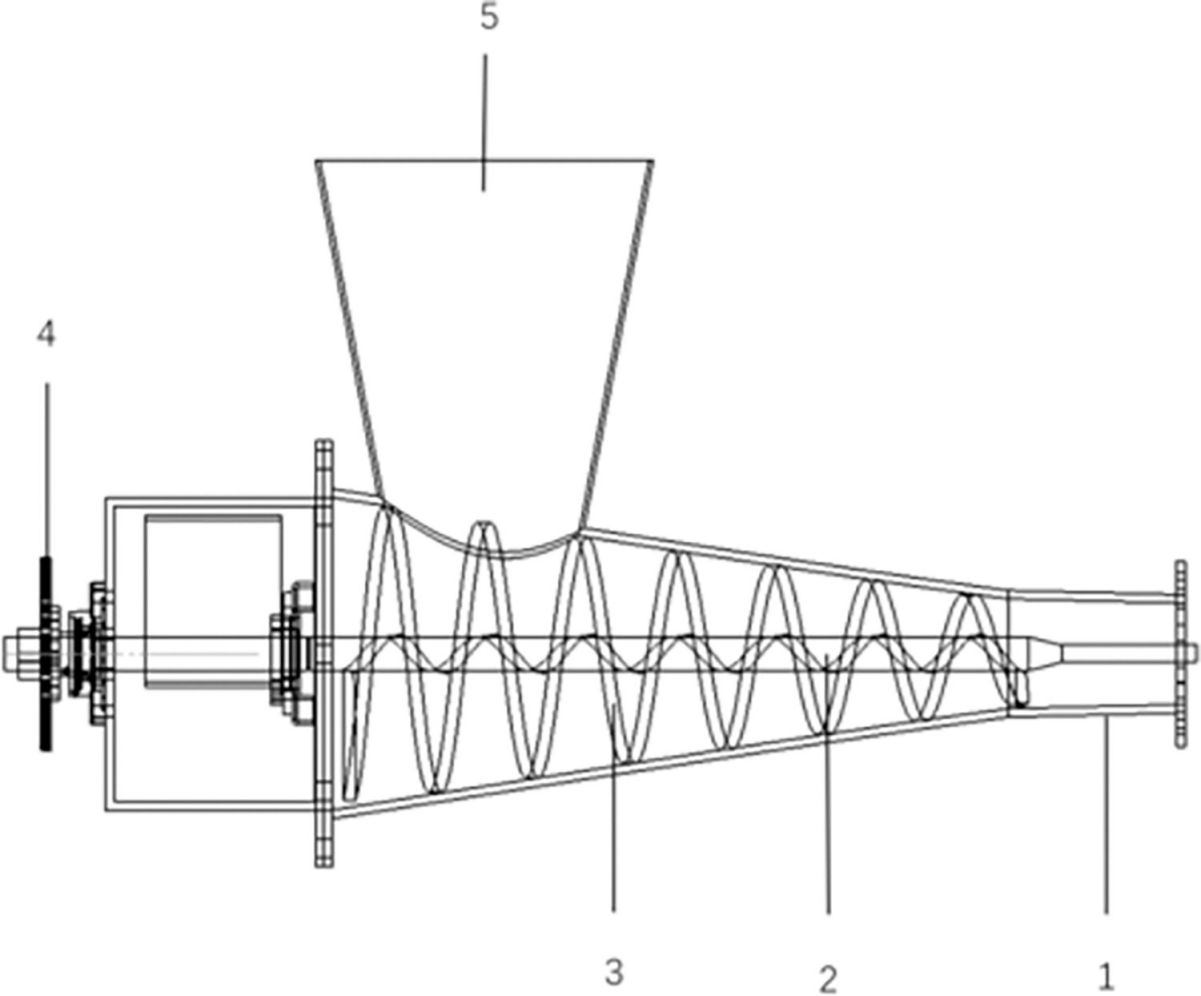
Forming device for reducing the diameter of the winch blades. 1. shell 2. winch shaft 3. winch blade 4. chain drive 5. feed port.

The forming device mainly consists of a feeding port, a winch shaft, a winch blade, and a machine shell. As shown in Fig 2, the transmission mode of the forming device is a chain drive. The working process of the corn straw composite pipe forming device is as follows: (1) Mixing of the mixture: by adding a certain proportion of water to cause even mixing of the straw and soil in the feed port to complete a mixing of the material and mixing the straw mixture of uniform material into the feed port. (2) Winch blade extrusion: The winch shaft drives the winch blade to rotate, and the winch blade extrudes and mixes with straw and soil in the process of rotating such that the materials are fully contacted and mixed to provide sufficient conditions for the molding of the composite pipe. (3) Outlet compression: The mixed material is extruded from the barrel, which is connected to the shell through the flange; thus, outlets with different outer diameter sizes were easily changed.

To ensure that the full amount of straw returned to the field, corn stover composite tube—forming equipment was applied every 1 meter forward, and the winch blade reducer molding device extruded the straw extruded into a compactness of 1.92 g/cm^3^, with a length of 1 meter using a compression ratio of 4:1 for the composite tube.

The maize planting pattern in the experimental field was 60 cm equidistant rows and 25 cm equidistant spacing, and the number of maize plants was calculated to be 66,667 plants/hm^2^. Through the method of weight measurement and averaging for the sampling of multiple stover plants in different areas, the mass of each stover plant was measured to be 698.276 g when the moisture content of the maize stover was 70.05%; this was calculated to be 4,655 g of stover plant per square meter.

According to the field measurements, the thickness of the straw covered by the corn combine harvester is approximately 2 cm, and the volume of the straw in the field with a width of 200 cm and a length of 100 cm is 4×10^4^ cm^3^. The working width of the field returning machinery is 200 cm, and the mass of the straw picked up by advancing 100 cm is 9,314 g. According to the compactness of 1.92 g/cm^3^, the volume of the straw in the shape of a round tube after compression is calculated and determined to be 4851 cm^3^. When the field return equipment advances 100cm, the length of the material is also 100 cm. According to the volume Formula v = πr^2^h (v = 4851 cm^3^, h = 100 cm), the radius (r) of the material outlet is 3.93 cm.

The molding device adopts the stepped continuous compression method for straw compression, which is divided into 6 compression spaces, with each space compressed in accordance with a 2:1 compression ratio. Considering the length of the compression bin and the assembly in the field return machine, the length of the compression bin was set at 81 cm according to the compression ratio relationship Eq (2) and the maximum screw diameter of the blade was determined to be 32.7 cm.


$$\nu =\frac{1}{3}\pi h(R^{2}+Rr+r^{2})$$ (2)

Eq: ν is the volume (cm^3^);

π is PI;

h is the horizontal distance of the compressed space, that is, the pitch (cm);

R is the large circle radius in compressed space (cm);

r is the small circle radius in compressed space (cm).

## Discrete element simulation analysis of the two forming devices

Discrete element simulation models of the two forming devices were established, and a simulation test was carried out. By studying the influence of the change in diameter of the spiral blade on the forming rate of the composite pipe and the output power of the auger shaft, the optimal corn stalk forming device could be determined.

### Three-dimensional modeling of molding devices

**Fig 3:**
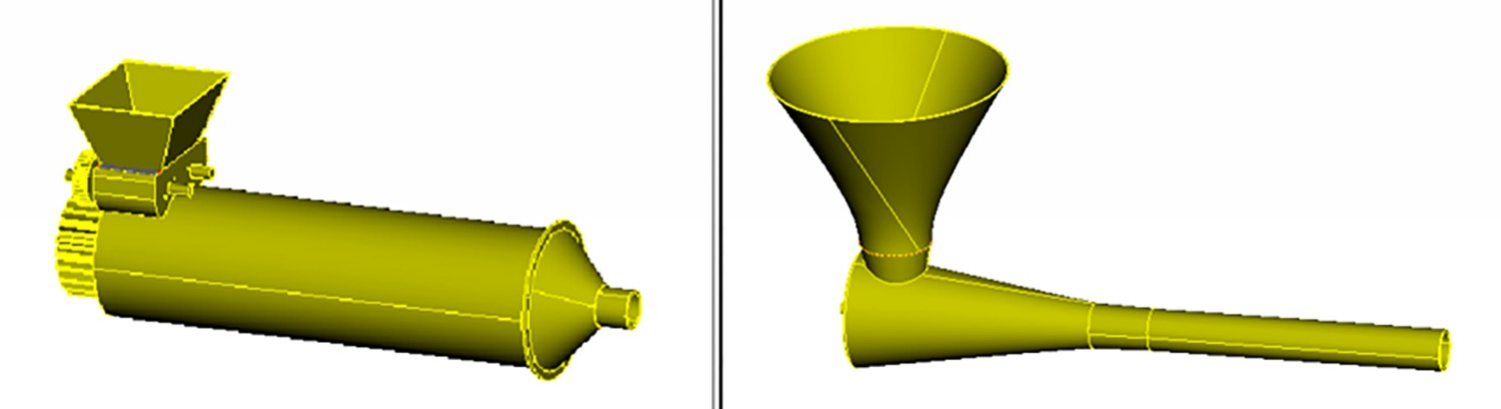
Simulation models of the two forming devices.

A three-dimensional model is established. To determine the working performance of the two forming devices, these models are imported into discrete element software, as shown in Fig 3.

### Determination of the simulation parameters

To establish the simulation model, the basic particle model needs to be defined, and the corn straw and soil particle models need to be created. The particles are composed of one or more spherical surfaces. The corn straw particles are composed of nine spherical surfaces, and the soil particles are represented by a spherical surface. A discrete element model of the composite pipe forming device is established, and the material property is given as steel. The spiral axis dynamics are specified, and the partial movement of the geometry is set during the simulation process. In this model, the corn straw particles and soil particles complete the extrusion-forming movement with the clockwise rotation of the screw shaft. The rotation of the screw shaft is set by the linear rotation power of the screw part. The rotation speed of the screw shaft is set to 470 rpm, and the direction and starting and ending points of the rotation shaft are set. The starting point is aimed at the end point along the radian direction of the spiral blade. According to the right-hand rule, the direction of the starting point pointing to the end point is the rotation direction of the spiral shaft, and the direction of the thumb is the discharge direction of the extruded composite pipe. A particle factory is created. The purpose of the particle factory board is to define the time, place, and method of the corn straw and soil particle models in the simulation. The parameters required for the simulation are shown in Table 1 [20–23].

**Table 1:** Simulation parameters.

parameter	numerical value	unit	parameter	numerical value
Device model density *ρ*_1_	7850	kg/m^3^	Soil-Soil recovery coefficient e_1_	0.25
Device model shear modulus G_1_	7.90×10^10^	Pa	Soil-Steel recovery coefficient e_2_	0.5
Device model Poisson’s ratio *ν*_1_	0.3		Straw steel recovery coefficient e_3_	0.6
Soil particle model bulk density *ρ*_2_	1385	kg/m^3^	Soil-Soil static friction coefficient *μ*_11_	0.7
Shear modulus of soil particles G_2_	2.97×10^8^	Pa	Soil-Steel static friction coefficient *μ*_12_	0.5
Poisson’s ratio of soil particles *ν*_2_	0.3		Straw-Steel static friction coefficient *μ*_13_	0.3
Straw particle model density *ρ*_3_	100	kg/m^3^	Soil-Soil rolling friction coefficient *μ*_21_	0.03
Shear modulus of straw particles G_3_	1×10^6^	Pa	Soil-Steel rolling friction coefficient *μ*_22_	0.01
Poisson’s ratio of straw particles *ν*_3_	0.4		Straw steel rolling friction coefficient *μ*_23_	0.01

**Fig 4:**
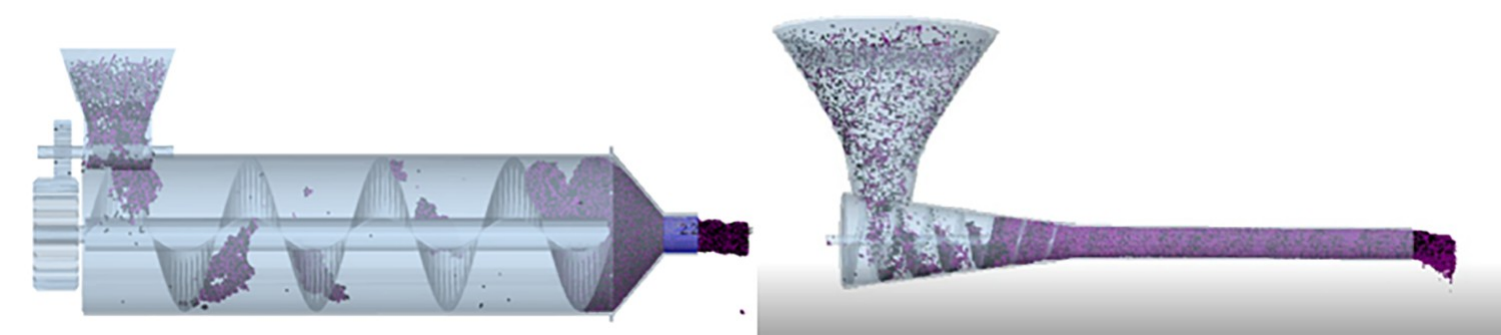
Simulation process of the two forming devices. (a) Spiral blade with no change (b) Spiral blades with change.

All particle plant plates need to be built on the geometric part. To sufficiently simulate the working state of the composite pipe forming device, a virtual plate is created at the top of the feed cylinder; this plate defines the area where the particles are generated in the particle model. Next, the domain of the simulation area is defined. The domain is the area where the simulation process will occur. The simulation simulator stops tracking any particles that are removed from the domain during the simulation process. The domain size has an effect on the simulation time; specifically, a larger domain correlates to a longer time required for simulation operation. The size of the calculation domain is based on the default size of the model of the imported composite pipe forming device [24–26]. The simulation process is shown in Fig 4.

### Simulation results and analysis

In the simulation test, to ensure the reliability of the test results, the forming mass flow rate of the composite pipe in a certain time period was used as the test index. Too little power during the working process could affect the operating efficiency of the equipment, and too much could increase its energy consumption. Therefore, the power needed to be evaluated reasonably and accurately. The output power of the two composite pipe-forming devices was obtained as a reference for the equipment evaluation.

#### Composite pipe forming rate

**Fig 5:**
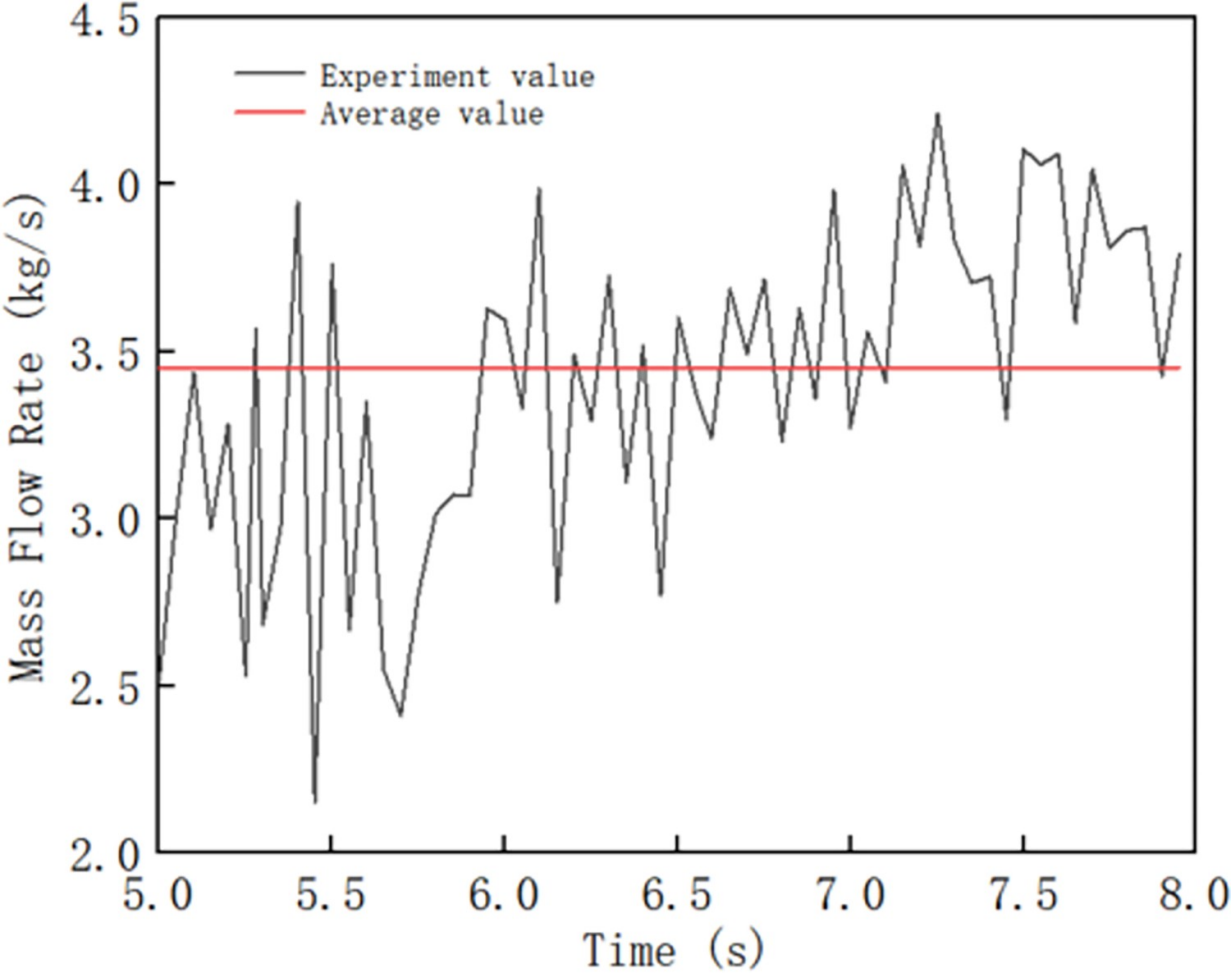
Forming rate of the composite tube of the winch blade without a reducer.

In Fig 5, the horizontal coordinate represents the simulation time (s), the vertical coordinate represents the molding rate (kg/s), and the red straight line represents the average value of each inflection point. To simulate the accuracy of the results, the simulation time was set to 5–8 s, the molding rate is the average mass flow rate of the composite pipe molded from the 5th to the 8th second, and the forming rate of the composite pipe of the winch blade without a reducer was 3.45 kg/s.

**Fig 6:**
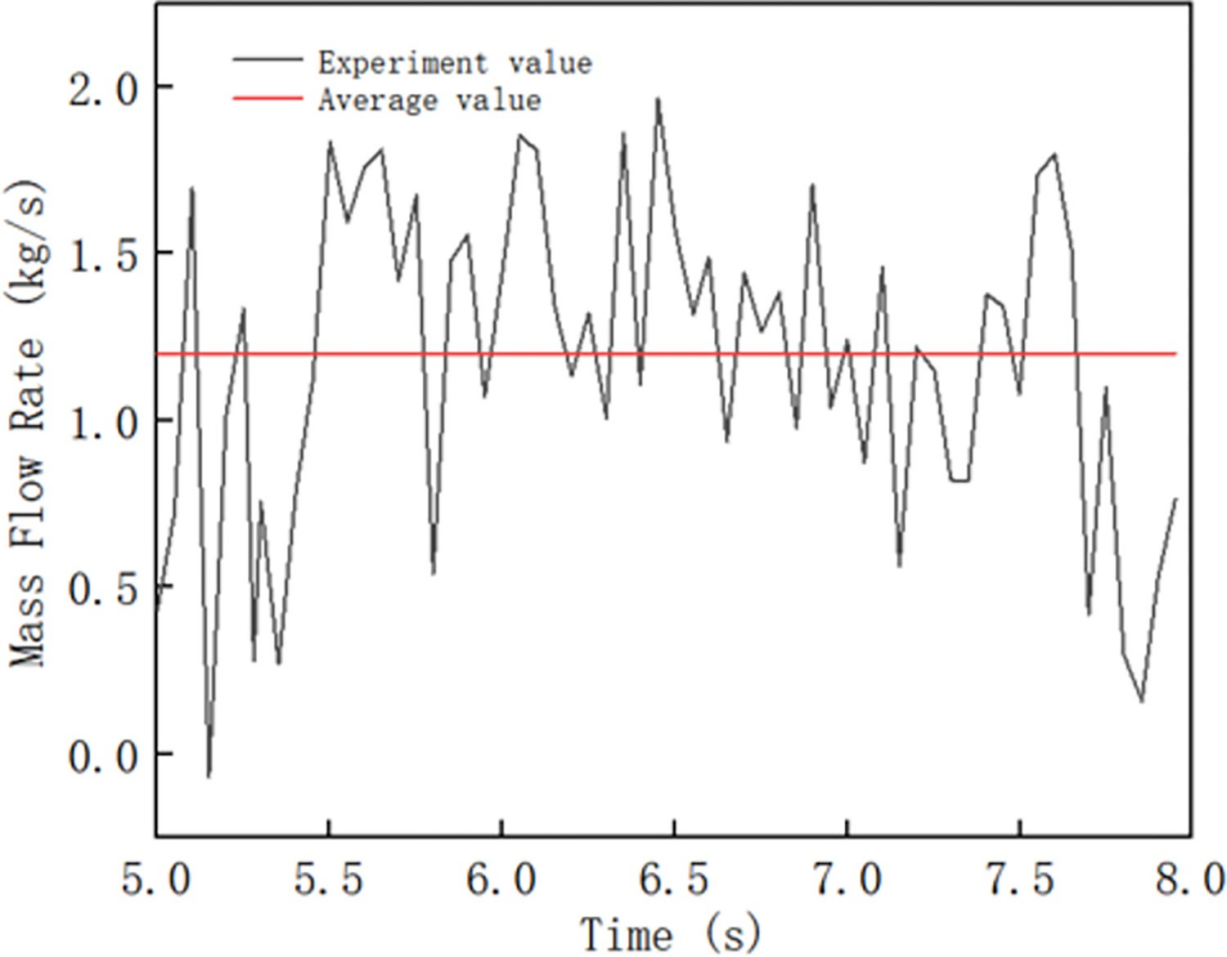
Forming rate of composite tubes with variable diameters of the winch blades.

In Fig 6, the abscissa represents the simulation time (s), the ordinate represents the forming rate (kg/s), and the red line represents the average value of each inflection point. To verify the accuracy of the simulation results, the simulation time was selected as 5–8 s, and the forming rate is the average mass flow rate of the composite pipe formed from the 5th second to the 8th second. The forming rate of the composite pipe with the spiral blade changing device was 1.20 kg/s.

#### Output power of the winch shaft

**Fig 7:**
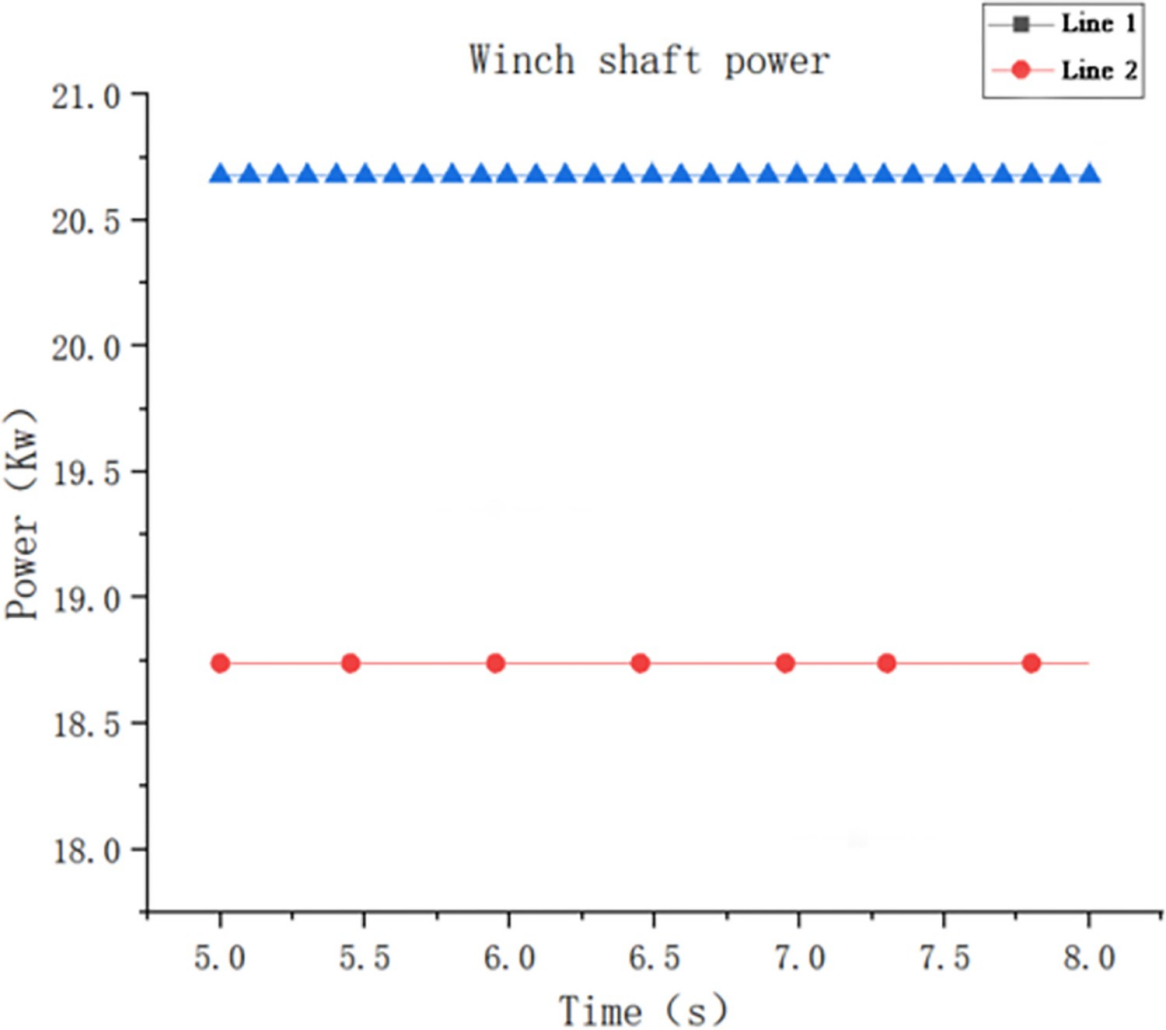
Output power of the winch shaft from the two different forming devices.

As shown in Fig 7, the abscissa represents the simulation time (s), the ordinate represents the output power of the keel shaft (kW), the green line 1 represents the output power of the winch shaft of the forming device without diameter change, and the red line 2 represents the output power of the winch shaft of the forming device with diameter change. For the accuracy of the simulation results, the simulation time is selected as 5–8 s, and the output power of the winch shaft is the average value from the 5th second to the 8th second. The output power of the winch shaft without the blade diameter change device was 20.7 kW, and the output power of the winch shaft with the blade diameter change device was 18.75 kW.

#### Simulation result analysis

Based on the above analysis, the best combination of composite pipe forming test devices on the composite pipe forming rate and the output power of the winch shaft need to be determined to improve the performance of the forming test device; combined with straw crushing and ditching buried equipment to form joint operation machinery, the above two indicators need to be normalized and analyzed. The comprehensive performance index is the weighted average of the two indices of the composite tube forming rate and the output power of the winch shaft according to a weight of 6:4; this value comprehensively reflects the performance of the forming test device, as shown in Table 2.

**Table 2:** Normalized calculation of the comprehensive performance of test indices.

Corn straw composite pipe forming device	Measured calculated value		normalized value		integrated performance index
	q/(kg⋅s^-1^)	w/(kW)	q/(kg⋅s^-1^)	w/(kW)	
No change	3.45	20.7	0.307317073	-0.310766722	0.060083555
Some changes	1.2	18.75	0.292682927	-0.333200732	0.042329463

Note: q is the discharge rate, kg⋅s^-1^; w is the power, kW; q is the normalized discharge rate; and w is the normalized power.

As shown in Table 2, through the weighted average of the 2 indicators of the composite pipe forming rate and winch shaft output power with different weights, the comprehensive performance index of the composite pipe forming device without a variable diameter was greater than that of the composite pipe forming device with a variable diameter; therefore, the winch blade without a variable diameter composite pipe forming device was more conducive to improving the composite pipe forming performance.

## Composite pipe forming test with two forming devices

To verify the overall working performance of the corn straw composite pipe forming device, a bench test was carried out at the test base of Moersite Agricultural Equipment Co., Ltd., in Hebi city, Henan Province. The forming density and forming rate of the composite pipe were used as test parameters to evaluate the working performance of the two forming devices.

### Test method

The test materials included maize straw, soil, and water, and the maize variety used was Zhonghe 170. The main equipment used for the test consisted of two kinds of corn straw composite pipe-forming devices: a motor, a speed control mixture material conveyor belt, a rapid moisture analyzer, an electronic scale, a stopwatch, a frequency converter, a tachometer, a ruler, and other measuring instruments.

After the corn harvest, the corn stalks were crushed and collected by a crusher near the base. The crushed corn stalks were then mixed with the soil in a certain proportion and weighed using an electronic scale. The mixture was stirred, and water was added to achieve a moisture content in the range of 20% to 26%. The density of the corn straw composite pipe should not be less than 1.30 g/cm^3^ to meet the forming performance index. The moisture content of the mixture was measured using a rapid moisture meter, and water was added and stirred until the moisture content met the test requirements.

**Fig 8:**
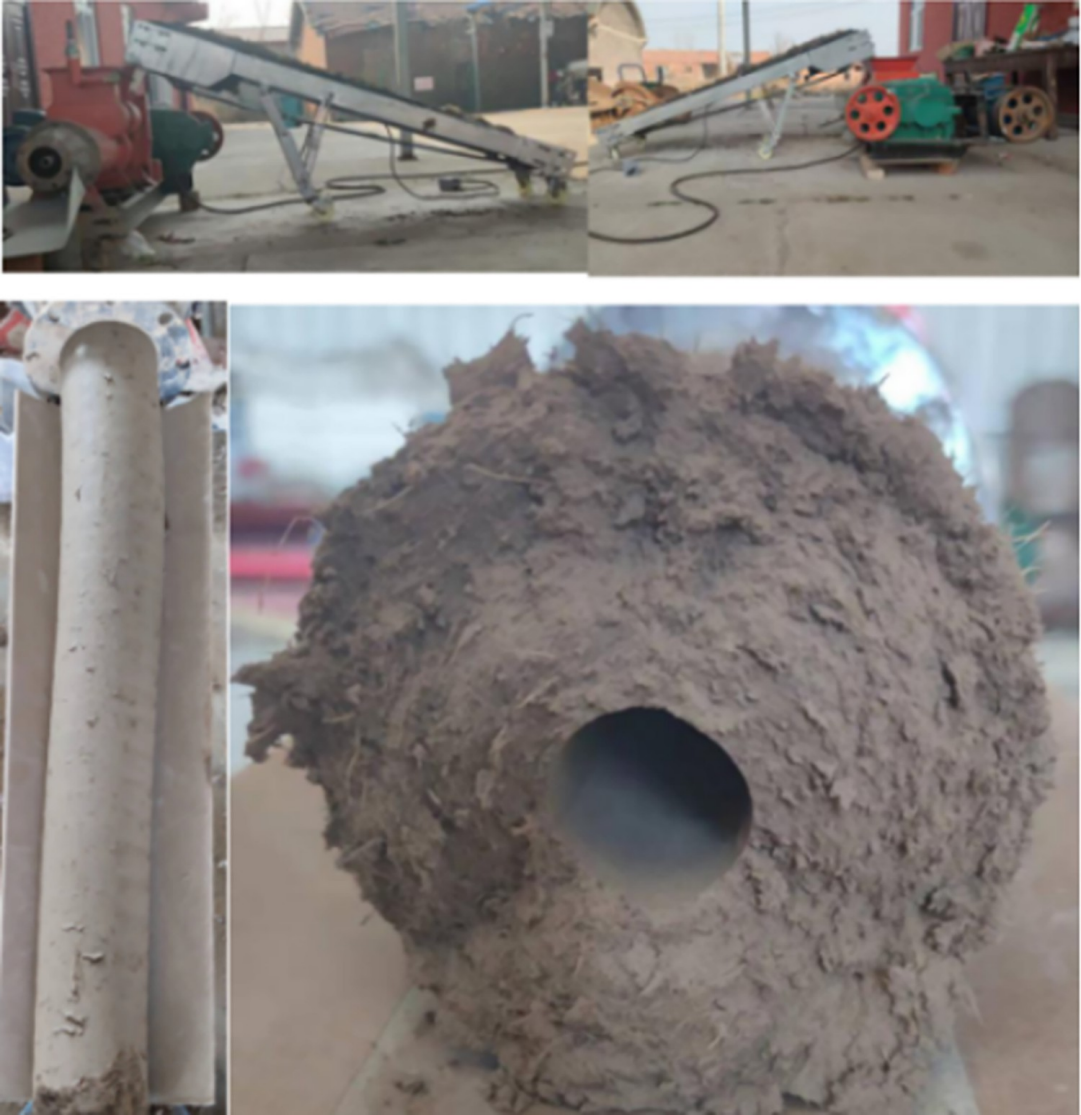
Bench test and formed composite pipe.

After the motor is energized and begins to rotate, the equipment is driven by chain and gear transmission, and the mixture material is transported to the feed port of the corn straw composite pipe forming device at the set conveying speed. Then, the mixture is sent to the equipment and compressed into tubes by stirring. During the extrusion process, a stopwatch is used to record the time required for the conveyor belt to transport the mixture and compress it into a tubular shape. A frequency conversion driver is used to adjust the speed, a tachometer is used to measure the speed of the winch shaft, and measuring tape is used to measure the length of the composite pipe. The bench test diagram and the resulting composite pipe are shown in Fig 8.

### Test results and analysis

(1) Determination of the forming density of the composite pipe
  According to previous tests, the outer diameter of the composite pipe is 100 mm, the inner diameter is 30 mm, and the forming density of the composite pipe is not less than 1.30 g/cm^3^. The 100-cm composite pipe was weighed, the forming weights of the six groups of composite pipes were recorded, and the forming density of the composite pipe was calculated. The forming density calculation of the composite pipe is shown in Formula (3).
$$\rho =\frac{m}{v}$$ (3)
  Eq: ρ is the degree of compaction (g/cm^3^);
  M is the composite pipe quality (g); and
  v is the composite pipe volume (cm^3^).
Determination of the forming rate of the composite pipe
  The forming time (s) of the corn straw composite pipe with a length of 100 cm was recorded by a stopwatch, and the forming rate (kg/ s) of the composite pipe was obtained by dividing the mass (kg) and forming time of the corn straw composite pipe with a length of 100 cm.

The average value of six bench tests was taken, and the test results are shown in Table 3.

**Table 3:** Test results.

serial number	No change		Some changes	
	forming density/(g/cm^3^)	forming rate/ (kg/s)	forming density/(g/cm^3^)	forming rate/ (kg/s)
1	1.87	3.12	1.56	1.99
2	1.91	3.11	1.43	2.35
3	1.93	3.13	1.54	1.84
4	1.94	3.28	1.39	1.76
5	1.94	3.41	1.48	2.33
6	1.94	3.32	1.57	2.12
experiment average	1.92	3.23	1.50	2.07

Table 3 shows that for the two kinds of forming devices, the composite tube density test average values of 1.92 g/cm^3^ and 1.50 g/cm^3^ are greater than 1.30 g/cm^3^ and meet the requirements of composite tube molding. With no decrease in the diameter molding device composite pipe forming rate, the average value is 3.23 kg/s, while with a decrease in the diameter molding device composite pipe forming rate, the average value is 2.07 kg/s, and the diameter of the molding device composite pipe forming rate is not reduced. These findings indicate that the use of a stranded blade without reducing the diameter of the composite pipe forming device is more conducive to improving the performance of composite pipe forming.

## Conclusion

To promote the comprehensive utilization of corn stover and the development of field water-saving irrigation technology, a method of returning corn stover to the field by crushing the crop stalks is proposed; in this method, the crush stalks are mixed with the soil in different proportions and extruded to form a hollow round tube. To compare the effect of the variation in the diameter of the stringer blade on the composite pipe forming performance, two composite pipe forming devices were theoretically designed; additionally, discrete element simulation software was used to simulate and analyze the composite pipe forming rate and the stringer shaft output power of the two forming devices. Based on the weighted average of these two indicators with different weights, the comprehensive performance indicators of the composite pipe molding device without a variable diameter was greater than those of the composite pipe molding device with a variable diameter. Therefore, a composite pipe-forming device without a variable diameter is more conducive to improving the composite pipe-forming performance.

According to the results from the corn stover composite pipe forming test, the composite tube density averages for an outer diameter of 100 mm and an inner diameter of 30 mm are greater than 1.30 g/cm^3^ and meet the requirements of composite tube molding; for a stranded blade without a reducer molding device, the composite tube molding rate average is 3.23 kg/s; for a stranded blade without a reducer molding device, the composite tube molding rate average is 2.07 kg/s; and for a composite pipe without a reducing device, the forming rate of the composite pipe of a of a stranded blade is faster. Thus, the forming device of a stranded blade without a reducing device is more conducive to improving the composite pipe molding performance.
